# Pseudotumor Cerebri with Blindness

**DOI:** 10.7759/cureus.13198

**Published:** 2021-02-07

**Authors:** Myoung Kwak, Gerald T Delk, Trilok Stead, Latha Ganti

**Affiliations:** 1 Emergency Medicine, Coliseum Medical Centers, Macon, USA; 2 Emergency Medicine, Trinity Preparatory School, Winter Park, USA; 3 Emergency Medicine, Envision Physician Services, Plantation, USA; 4 Emergency Medicine, University of Central Florida College of Medicine, Orlando, USA; 5 Emergency Medicine, Mercer University School of Medicine, Macon, USA

**Keywords:** pseudotumor cerebri, idiopathic intracranial hypertension

## Abstract

Pseudotumor cerebri, or idiopathic intracranial hypertension (IIH), is a syndrome of elevated intracranial pressure (ICP) of unknown etiology that occurs predominantly in obese women of childbearing age. Pseudotumor cerebri literally means "false brain tumor". It is a “diagnosis of exclusion” therefore a complete work-up to rule out life-threatening causes for increased ICP must be performed through a comprehensive history, complete physical examination, diagnostic imaging, and cerebrospinal fluid (CSF) analysis before the diagnosis can be made. The authors present the case of a young woman with headache, and near blindness due to pseudotumor cerebri. The presentation, diagnosis, and treatment options are discussed.

## Introduction

Pseudotumor cerebri is a condition characterized by elevated intracranial pressure (ICP). It is predominantly seen in overweight women of childbearing age [1]. Obesity is both a contributing factor as well as a therapeutic target. Weight loss is an effective treatment [2]. The precise pathogenesis is not known [3], and hence it is also known as idiopathic intracranial hypertension (IIH). It can manifest as a variety of symptoms, including headache, visual disturbances, and tinnitus. Visual disturbances include diplopia, visual field defects, scotomas, extraocular movement dysfunction (especially CN 6), and even vision loss. Symptoms are alleviated by decreasing ICP. Treatment can encompass pharmacologic or surgical means. Surgical interventions include cerebrospinal fluid (CSF) diversion techniques such as optic nerve sheath fenestration (ONSF), bariatric surgery, and venous sinus stenting [4].

## Case presentation

A 26-year-old female with a past medical history significant for hypertension and chronic renal insufficiency treated without hemodialysis presented to the emergency department with moderate to severe headaches associated with bilateral visual disturbances. The onset was nine days prior to presentation and was described as a right frontal headache with gradual loss of vision in the right eye, followed by vision changes in the left eye. She reported seeing only hand movement with the right eye, and finger counting only with the left eye. She also had neck pain, chest pain, numbness in both hands, and mild shortness of breath, but denied any other symptoms. Specifically, she denied fever, chills, recent injury, numbness, weakness, or undergoing any spinal procedure. She also acknowledged she went to a hospital out of state a few days prior to the presentation where was evaluated for a “stroke” and confirmed she did not have one and received a CT scan of the brain. On the day of presentation, she noted her left-sided vision was getting worse, so she decided to get a second opinion. Her mother was noted to have had similar symptoms when she was 28 and was diagnosed with a “stroke,” and had received spinal taps. 

On physical examination, the patient was found to be morbidly obese with a body mass index (BMI) of 38. Her initial vital signs were blood pressure 196/141 mmHg, pulse 80 beats per minute, respirations 16 breaths per minute, temperature of 36.8° C, and oxygen saturation of 98% on room air. She could not read the top line of the eye chart. The vision in her right eye was grossly reduced to hand waving only. She had light perception, an afferent pupillary defect, and abducens nerve palsy. The left eye vision was grossly reduced to finger counting only. Extraocular movements were intact. There was a mild proptosis. The pupil was mildly dilated and sluggish with light reflex. She had difficulty tracking the examiner's finger. Disorders of conjugate gaze and uncoordinated eye movement were noted bilaterally. Fundoscopic exam revealed gross papilledema bilaterally. There was pain associated with the headache, but no redness or injection of the eye. The rest of the physical examination was unremarkable. Specifically, her neurologic examination (strength, sensation, reflexes, and remainder of cranial nerves) and mental status were unremarkable.

Laboratory analyses including complete blood count and electrolyte panel were unremarkable. CT of the brain without contrast and MRI of the brain without contrast were negative. Due to the clinical history and examination along with the unremarkable findings, a lumbar puncture was performed. The opening pressure was noted to be 52 cmH_2_O. The CSF was clear, colorless, with zero white or red blood cells, glucose 62 mg/dL, and total protein 23.0 mg/dL. The patient noted some improvement of the headache following the lumbar puncture, but there was no change with the visual deficits. Repeat lumbar puncture was done and the opening pressure was noted to be 10. The patient was diagnosed with IIH producing significant visual impairment and right abducens nerve palsy. The patient was transferred to a higher echelon of care for emergent ONSF.

## Discussion

Pseudotumor cerebri or IIH is a pathologic disorder affecting obese women of childbearing age that, if left untreated, can lead to vision loss. The main goals of treatment include early identification, alleviation of symptoms (headache, visual disturbances), and preservation of vision. This disease process is a diagnosis of exclusion, therefore, other causes of increased ICP need to be excluded with brain with imaging (ideally MRI brain) and lumbar puncture before the diagnosis can be confirmed. The Modified Dandy Criteria for IIH [5] can be helpful in making the diagnosis (Table 1).

**Table 1:** Modified Dandy Criteria for idiopathic intracranial hypertension (IIH) CSF: cerebrospinal fluid

Table 1: Modified Dandy Criteria for IIH
Presence of signs and symptoms of increased intracranial pressure
Absence of localizing findings on neurologic examination except those known to occur from increased intracranial pressure
Absence of deformity, displacement, or obstruction of the ventricular system and otherwise normal neurodiagnostic studies, except for evidence of increased cerebrospinal fluid pressure (> 200 mmH2O).
Abnormal neuroimaging except for empty sella turcica, optic nerve sheath with filled out CSF spaces, and smooth-walled non flow-related venous sinus stenosis or collapse should lead to another diagnosis
Awake and alert patient
No other cause of increased intracranial pressure present

The differential diagnosis includes side effects from certain medications (such as tetracyclines, and lithium) and cerebral venous sinus thrombosis. The later diagnosis has an indistinguishable clinical presentation and must be excluded before IIH can be diagnosed. If the headaches are the result of elevated ICP, then the headache can improve after a lumbar puncture is performed. However, many patients have other coexisting mixed headache disorders and headache response after the LP does not necessarily rule in or rule out the diagnosis [1].

Weight loss is routinely recommended for all IIH patients due to its association with obesity. In patients with minimal symptoms, signs, and visual loss, a weight management program with a low-salt diet and lifestyle changes, including an exercise program, is a reasonable initial treatment strategy. However, weight loss alone may be inadequate [1].

A variety of medical treatments can be considered when symptoms and visual disturbances are mild (Table 2) [1]. The preferred drug is acetazolamide. Other medical treatments (topiramate, furosemide, or prednisone) can be added or substituted when acetazolamide is insufficient as monotherapy, or the medication is poorly tolerated.

**Table 2:** Medications used for idiopathic intracranial hypertension (IIH) CSF: cerebrospinal fluid

Table 2: medications used for pseudotumor cerebri/idiopathic intracranial hypertension		
Drug	Mechanism of Action	Adverse Effects
Acetazolamide	Inhibition of carbonic anhydrase, decrease CSF production	Dizziness, increased urination, blurred vision, dry mouth, nausea, vomiting, diarrhea, etc.
Topiramate	Weak carbonic anhydrase inhibitor, blockade of voltage-dependent sodium and calcium channels, inhibits glutamate pathway	Dizziness, anxiety, fatigue, diarrhea, weight loss, etc.
Furosemide	Loop diuretics	Increased urination, low blood pressure, dehydration, electrolyte depletion, muscle cramps, dizziness, diarrhea, etc.
Prednisone	Unclear	Aggression, agitation, blurred vision, dizziness, headache, weight gain, rebound intracranial hypertension

Acetazolamide, a carbonic anhydrase inhibitor, is the initial treatment of choice and thought to decrease CSF production. The subsequent decrease in ICP often leads to improved symptoms. Topiramate is most commonly used as an adjunct treatment of the various associated headache disorders. For severe or progressing visual field, surgical treatments that include ONSF or CSF shunting are recommended to prevent irreversible visual loss.

During diagnostic work-up for IIH, headache symptoms often improve following the diagnostic lumbar puncture, but this improvement is usually transient. Repeat lumbar punctures are no longer considered standard treatment due to technical difficulties and patient tolerance. Nevertheless, a lumbar puncture can be a useful temporizing measure in patients with an acute exacerbation of symptoms or a fulminant presentation [6].

CSF diversion (e.g., ventriculo-peritoneal and lumbo-peritoneal shunting) and ONSF are the commonly used surgical treatments. The choice of procedure depends on consultant training and expertise, but ONSF may be preferred when papilledema associated vision loss is the primary issue. However, CSF diversion is preferred when patients have significant symptoms associated with papilledema and visual loss [7].

CSF diversion through shunting rapidly reduces ICP leading to improvement in signs and symptoms of pseudotumor cerebri. In patients with moderate to severe symptomatology and no papilledema, CSF diversion should not be performed because the reduction in ICP will not likely improve the associated headache [8]. ONSF, on the other hand, improves visual symptoms by directly reducing the pressure on the optic nerve head without decreasing ICP. Although papilledema and visual loss in the other eye can improve, patients with bilateral visual loss require bilateral sequential ONSF. ONSF should not be performed if there is no papilledema or if visual field loss is minimal [9].

## Conclusions

Pseudotumor cerebri (IIH) is a diagnosis of exclusion but an early diagnosis can help alleviate symptoms and mitigate against vision loss. Treatment includes weight loss through dieting, lumbar puncture, pharmacological agents, and surgical interventions designed to decrease papilledema or the elevation in ICP as the result of CSF build-up.
